# Predicting Acute Viral Hepatitis Serum Markers (A and E) in Patients with Suspected Acute Viral Hepatitis Attending Primary Health Care Centers in Baghdad: A One Year Cross-Sectional Study

**DOI:** 10.5539/gjhs.v4n5p172

**Published:** 2012-08-21

**Authors:** Ahmed Samir Al-Naaimi, Atallah Mekhlef Turky, Hanan Abdulghafoor Khaleel, Rasha Waleed Jalil, Olah A. Mekhlef, Susan Abdul Kareem, Nadia Yousif Hasan, Azhar Abdulla Dhadain

**Affiliations:** 1 Baghdad Medical college, Baghdad University, Baghdad, Iraq; 2 Viral Hepatitis Section, Communicable Disease Control Center, Public Health Directorate, Ministry of Health, Baghdad, Iraq; 3 Central Public Health Laboratories, Public Health Directorate, Ministry of Health, Baghdad, Iraq

**Keywords:** anti HAV IgM, anti HEV IgM, acute viral hepatitis, Baghdad

## Abstract

**Background:**

Viral hepatitis is an important preventable infectious disease with various rates of occurrence in different areas of the world. The main objective of the present study was to evaluate the role of some risk factors in predicting a positive acute viral hepatitis marker among patients with suspected acute viral hepatitis in a primary health care setting in Baghdad. Besides, finding out the occurrence of jaundice, contribution of viruses A and E to the cases that have occurred in Baghdad province was also searched for.

**Methods:**

Over a period of 1 year a descriptive cross sectional study was carried out at the primary health care centers in Baghdad. A questionnaire form was used to collect data about demographic factors and the results of the investigations. Total serum bilirubin and bilirubin in urine were done at the primary health care center laboratory. The rest of the sera samples were sent to Hepatitis referral Lab at Central Public Health Laboratory (CPHL) to be tested for anti HAV IgM and anti HEV IgM using ELISA technique.

**Results:**

A total of 7,576,372 consultations to primary health care centers were recorded in Baghdad. Among those a total of 2,692 cases (35.5 per 100,000 consultations) were labeled as acute viral hepatitis cases. A positive hepatitis viral marker (A, B, C and E) was found in 1,332 cases (17.6 per 100,000 consultations). More than two fifths (44.8%) of cases were positive for anti-HAV antibodies and another 1.6% had positive anti-HEV antibodies.

**Conclusion:**

During 1 year period, the rate of occurrence of suspected acute viral hepatitis cases was 35.5 per 100000 of consultations to the primary health care centers in Baghdad. Of the total suspected cases, only 17.6 per 100000 of the consultations were positive for one of the viral hepatitis markers. Those who tested positive for one of the viral hepatitis markers represent 49.5% of the suspected cases. Proportion of anti HAV IgM positive tests among suspected cases was 44.8%. Factors that were able to predict positive Anti HAV IgM were (younger age group, negative history of cupping or tattooing, negative past history of jaundice). Male gender was the least important predictor of a positive case for anti HAV IgM. Proportion of Anti HEV IgM positive tests among suspected cases was 1.6%. Of the factors studied, only age was able to predict a positive Anti HEV IgM in those more than 15 years. Further studies are recommended.

## 1. Introduction

Among several viruses that infect the liver causing hepatitis, there are distinct viruses which are grouped as the viral hepatitides; they are primarily hepatotrophic and they all have similar clinical presentations, but differ in etiology and in some epidemiological, immunological, clinical and pathological characteristics. Jaundice is an important and common feature of the illness since the liver is the principle organ affected (Heymann, 2004).

The identification of these viruses has started with the detection of Hepatitis B virus (HBV) in 1970, followed by Hepatitis A virus (HAV) in 1973, Hepatitis D virus (HDV) in 1977, Hepatitis E virus (HEV) in 1983, Hepatitis C virus (HCV) in 1989 and lastly Hepatitis G virus (HGV) in 1996 (Kuntz, 2008).

HAV is a ubiquitous virus, especially since it retains its stability in heat and cold under normal environmental conditions and is highly resistant to external influences. HAV infection is largely acquired through fecal oral transmission (Jeong, 2010). In highly endemic areas, most infections occur in early childhood and those infected may not experience any noticeable symptoms therefore epidemics are uncommon because older children and adults are generally immune (Kamal, 2010).

HAV is a ubiquitous virus, especially since it retains its stability in heat and cold under normal environmental conditions and is highly resistant to external influences. HAV infection is largely acquired through fecal oral transmission (Jeong, 2010). In highly endemic areas, most infections occur in early childhood and those infected may not experience any noticeable symptoms therefore epidemics are uncommon because older children and adults are generally immune (Kamal, 2010).

Hepatitis E Virus (HEV) infection occurs endemically and well as sporadically (because of travel) (Kuntz, 2008). This enterically transmitted virus is prevalent throughout much of the developing world. It is endemic in countries where human waste is allowed to get into drinking water without first being purified. Hepatitis E is a short lived, self-limiting viral infection followed by recovery. Prolonged viraemia and viral shedding are unusual and chronic infection does not occur (Kamal, 2010). Fulminant hepatitis occurs more frequently in pregnancy and induces a mortality rate of 20% and can also cause premature births (Meng, 2009) (FitzSimons, 2010).

The aim of this work is to evaluate the role of some risk factors in predicting a positive acute viral hepatitis marker among patients with jaundice in a primary health care setting in Baghdad.

## Objectives

1. Assess the occurrence rate of suspected acute viral hepatitis and positive viral hepatitis a marker among clients of primary health care centers in Baghdad.
2. Assess the proportion of positive hepatitis A and E viral marker from the total suspected cases.
3. Assess the contribution of 10 selected explanatory variables to the risk of testing positive for HAV and HEV serum markers. These factors are: History of blood transfusion, History of tattoo or cupping, Age group, Past history of jaundice, History of chronic illness, Past Surgical History, Past history of Dentist visit, Primary Health Care Department (Alrusafa compared to Al-Karkh), History of jaundice in the contacts and Gender (male compared to female).

## 2. Methods

### 2.1 Study Population

Patients attending the primary health care centers in Baghdad (Alrusafa and Alkarkh) with jaundice or signs and symptoms suggestive of acute viral hepatitis were included in the study.

### 2.2 Case Definition and Criteria of Inclusion

Patients with one or more of the following characteristics were included in the study:

1. Acute clinical illness that includes malaise, extreme fatigue, fever, anorexia, vomiting. Combined with right upper quadrant pain and dark urine.
2. Clinical jaundice and positive bile pigment in urine and elevated total serum bilirubin.
3. History of contact with an acute or known chronic case of viral hepatitis.

### 2.3 Study Sample

All eligible subjects recorded during the one year study period extending from 15^th^ March 2010 to 15^th^ March 2011 were included in the study.

### 2.4 Data Collection

A specially designed semi-structured questionnaire form was used to collect data about demographic factors like age, sex, and residence in addition to history of illness, possible risk factors and the results of the investigations.

Blood sample (5- 8 ml) was drawn from all patients as a part of routine laboratory work and sera were separated at the primary health care center laboratory in each sector. Total serum bilirubin and bilirubin in urine were done at the primary health care center laboratory. The rest of the sera samples were deep freezed and sent to the Central Public Health Laboratory (CPHL) to be tested for anti HAV IgM and anti HEV IgM using ELISA technique at the reference laboratory for viral hepatitis.

### 2.5 Ethical Considerations

The data collected for the purposes of current research were part of standard clinical practice of primary health care center when dealing with patients with suspected acute viral hepatitis. Patient’s privacy was secured and identifying information was kept confidential. Prompt treatment was provided for all study subjects.

### 2.6 Statistical Analysis

Epi info v.3.5.1 was used to enter the data and statistical analysis was done using SPSS v20. Odds ratio was used to present the strength of association between 2 dichotomous variables. Chi-square test was used to assess the statistical significance of cross-tabulation for categorical variables. Discriminant analysis was used to adjust for confounding effect when assessing the contribution of a list of explanatory variables to the risk of testing positive for a viral marker. P value less than 0.05 was considered statistically significant.

## 3. Results

A total of 7,576,372 consultations to primary health care centers were recorded in Baghdad. Among those a total of 2,692 cases (35.5 per 100,000 consultations) were labeled as suspected acute viral hepatitis. A positive hepatitis viral marker (A, B, C and E) was found in 1,332 cases (17.6 per 100,000 consultations). More than two fifths (44.8%) of jaundice cases with positive viral markers had anti-HAV antibodies and another 1.6% had positive anti-HEV antibodies in their tested sera, Table 1.

**Table 1:** Relative frequency of positive anti-HAV and HEV among suspected cases

Total suspected cases =2,692	N	%	95% confidence interval
Anti-HAV antibodies	1,206	44.8	(42.9% - 46.7%)
Anti-HEV antibodies	42	1.6	(1.1% – 2.1%)

### 3.1 Anti-HAV

The risk of testing positive for anti-HAV is highest in children less than 15 years of age. The risk in children was 17 times significantly higher than that in >45 years of age group. Males significantly increase the risk by 19% compared to females. A negative past history of jaundice significantly increase the risk of positive anti-HAV by almost 3 times. A negative history of jaundice in contacts significantly increases the risk by 34%. A negative history of blood transfusion was associated by a statistically significant two times increase in risk. A negative history of chronic illness significantly increases the risk by 2.3 times. A negative surgical history significantly increases the risk by 2 times. A negative history of tattoo or cupping significantly increases the risk by 7.3 times. A negative history of dentist visit significantly increases the risk by 1.91 times. Finally, being in Alkarkh sector is associated by a statistically significant increase in risk of 47% compared to Alrussafa sector, Table 2.

**Table 2:** The risk of positive anti-HAV antibodies by selected explanatory variables

	Total	Anti-HAV antibodies		OR	95% CI for OR	P
	N	N	%			
**Age groups**						<0.001
less than 5 yrs	406	262	64.5	17.37	(9.03 – 33.39)	<0.001
5-14 yrs	1186	761	64.2	17.09	(9.08 – 32.17)	<0.001
15-44 yrs	981	171	17.4	2.02	(1.06 – 3.83)	0.02
more than 45 yrs	116	11	9.5	Reference		

**Gender**						0.024
Female	1223	519	42.4	Reference		
Male	1466	686	46.8	1.19	(1.02 - 1.39)	

**Past history of jaundice**						<0.001
Negative	2315	1110	47.9	2.96	(2.27 - 3.87)	
Positive	329	78	23.7	Reference		

**History of jaundice in the contacts**						0.003
Negative	2105	980	46.6	1.34	(1.11 - 1.61)	
Positive	565	223	39.5	Reference		

**History of blood transfusion**						0.011
Negative	2609	1187	45.5	2.06	(1.17 - 3.64)	
Positive	59	17	28.8	Reference		

**History of chronic illness**						<0.001
Negative	2576	1179	45.8	2.30	(1.44 - 3.65)	
Positive	93	25	26.9	Reference		

**Past Surgical History**						<0.001
Negative	2443	1136	46.5	2.02	(1.5 - 2.72)	
Positive	223	67	30	Reference		

**History of tattoo or cupping**						<0.001
Negative	2602	1197	46	7.30	(3.33 - 16.04)	
Positive	67	7	10.4	Reference		

**Past history of Dentist visit**						<0.001
Negative	2214	1056	47.7	1.91	(1.54 - 2.38)	
Positive	437	141	32.3	Reference		

**Primary Health Care Department**						<0.001
Alkarkh	1199	600	50	1.47	(1.26 - 1.71)	
Alrusafa	1493	606	40.6	Reference		

Discriminant analysis was used to rank selected independent (explanatory) variables according to their capacity in predicting a specific positive viral hepatitis marker. It is a type of multivariate analysis, since the independent variables are entered together. The list of eligible predictors include: History of blood transfusion, History of tattoo or cupping, Age group, Past history of jaundice, History of chronic illness, Past Surgical History, Past history of Dentist visit, Primary Health Care Department (Alrussafa compared to Alkarkh), History of jaundice in the contacts and Gender (male compared to female).

As shown in Table 3, the predictors for positive serum anti-HAV antibodies are ordered according to their importance in predicting positive cases. A negative sign for the coefficient indicates that the presence of this factor is associated with more probability for the tested subject being positive for this viral hepatitis marker, while a positive coefficient indicates increased probability of being negative for this hepatitis marker. For example being a male will increase the probability of being positive for HAV (since the coefficient has a negative sign), while the remaining variables when positive (or older age) increase the probability of being negative for HAV antibodies.

**Table 3:** Discriminant analysis model with selected independent variables and results of serum anti-HAV antibodies test as the dependent variables

Predictors	Coefficient
Age group	0.916
Past history of jaundice	0.334
History of tattoo or cupping	0.237
Past history of Dentist visit	0.233
Primary Health Care Department	0.23
Past Surgical History	0.167
History of chronic illness	0.136
History of jaundice in the contacts	0.132
History of blood transfusion	0.093
Gender	-0.09

Note: *Coefficient: Pooled within-groups correlations between discriminating variables and standardized canonical discriminant functions.

From the list of 10 predictors used in the discriminant model only 6 were enough to yield a discriminant model which was able to predict positive anti-HAV with 73.5% accuracy and an overall prediction accuracy of 70.8%. The predictive accuracy for the positive group is as important as that of the negative group in this model, since the prevalence of anti-HAV antibodies in the overall sample was close to 50%, table 4.

**Table 4:** Forward selection method for Discriminant analysis model with selected independent variables and results of serum anti-HAV antibodies test as the dependent variables

Selected independent variables	Coefficients used in the discriminant equation
Age group (coded as 1 for <5 yrs, 2 for 5-14 yrs, 3 for 15-44 yrs and 4 for 45+ yrs)	1.253
History of tattoo or cupping(coded as 1 for positive and zero for negative)	0.746
Past history of jaundice(coded as 1 for positive and zero for negative)	0.738
Primary Health Care Department (coded as 1 for Alrusafa and zero for Alkarkh)	0.475
History of jaundice in the contacts(coded as 1 for positive and zero for negative)	0.322
Gender (coded as 1 for male and zero for female)	-0.204
(Constant)	-3.213

	**Discriminant score at group centroids**
Negative Anti-HAV antibodies	0.447
Positive Anti-HAV antibodies	-0.54
Critical value	-0.0465

P (model)<0.001

Discriminant score (D) = -3.213 + [1.253 × Age group (coded as 1 for <5 yrs, 2 for 5-14 yrs, 3 for 15-44 yrs and 4 for 45+ yrs)] - [0.204 × Gender (coded as 1 for male and zero for female)] + [0.738 × Past history of jaundice (coded as 1 for positive and zero for negative)] + [0.322 × History of jaundice in the contacts (coded as 1 for positive and zero for negative)] + [0.746 × History of tattoo or cupping (coded as 1 for positive and zero for negative)] + [0.475 × Primary Health Care Department (coded as 1 for Alrusafa and zero for Alkarkh)].

**Table:** 

Accuracy of prediction for negative test results	73.5%
Accuracy of prediction for Positive test results	67.5%
Overall Accuracy of prediction	70.8%

A younger age group was the most powerful predictor for positive HAV marker, followed by a negative history of tattoo or cupping and a negative past history of jaundice. A jaundice case examined in Alkarkh district and a negative history of jaundice in contacts contributed also to predicting a positive HAV case. Finally a male gender was the least important of the 6 model selected predictors in predicting positive case. When the value of these predictors is entered in the equation the discriminant score (D) is calculated. If the resulting D score for a subject is higher than the critical value of -0.0465 then we predict a negative HAV status, while a D score lower than the critical value predicts a positive HAV antibodies case. The more extreme is the calculated value of D the higher our confidence in the prediction accuracy, Table 4.

### 3.2 Anti-HEV

As shown in Table 5, only age showed a statistically significant positive association with the risk of having positive anti-HEV antibodies. Compared to children less than 5 years old in whom the rate of positive anti-HEV was lowest (0.5%), adults 15-44 years of age had the highest positivity rate (3%). This age group was associated with 6.15 times increase in the risk compared to less than 5 years of age children. The remaining explanatory variables showed no statistically significant association with the risk of testing positive for anti-HEV antibodies.

**Table 5:** The risk of positive anti-HEV antibodies by selected explanatory variables

	Total N	Anti-HEV N	Antibodies %	OR	95% CI for OR	P
Age groups						<0.001
less than 5 yrs	406	2	0.5	Reference		
5-14 yrs	1186	9	0.8	1.54	(0.33 – 7.18)	0.58[NS]
15-44 yrs	981	29	3	6.15	(1.46 – 25.91)	0.005
more than 45 yrs	116	1	0.9	1.76	(0.16 – 19.55)	0.64[NS]

**Gender**						0.51[NS]
Female	1223	17	1.4	Reference		
Male	1466	25	1.7	1.23	(0.66 - 2.29)	

**Past history of jaundice**						0.19[NS]
Negative	2315	34	1.5	Reference		
Positive	329	8	2.4	1.67	(0.77 - 3.64)	

**History of jaundice in the contacts**						0.97[NS]
Negative	2105	33	1.6	Reference		
Positive	565	9	1.6	1.02	(0.48 - 2.14)	

**History of blood transfusion**						0.94[NS]
Negative	2609	41	1.6	Reference		
Positive	59	1	1.7	1.08	(0.15 - 7.99)	

**History of chronic illness**						0.65[NS]
Negative	2576	40	1.6	Reference		
Positive	93	2	2.2	1.39	(0.33 - 5.85)	

**Past Surgical History**						0.05[NS]
Negative	2443	35	1.4	Reference		
Positive	223	7	3.1	2.23	(0.98 - 5.08)	

**History of tattoo or cupping**						0.35[NS]
Negative	2602	40	1.5	Reference		
Positive	67	2	3	1.97	(0.47 - 8.33)	

**Past history of Dentist visit**						0.20[NS]
Negative	2214	32	1.4	Reference		
Positive	437	10	2.3	1.60	(0.78 - 3.27)	

**Primary Health Care Department**						0.25[NS]
Alkarkh	1199	15	1.3	Reference		
Alrusafa	1493	27	1.8	1.45	(0.77 - 2.75)	

As shown in Table 6, the predictors for positive serum anti-HEV antibodies are ordered according to their importance in predicting positive cases. A negative sign for the coefficient indicates that the presence of this factor is associated with a higher probability for the tested subject being negative for this viral hepatitis marker, while a positive coefficient indicates increased probability of being a case. For example having a positive history of jaundice in contacts will reduce the probability of being positive for HEV, while the remaining variables when positive (in addition to being a male or older age) increase the probability of being an HEV positive case.

**Table 6:** Discriminant analysis model with selected independent variables and results of serum anti-HEV antibodies test as the dependent variables

Predictors	Coefficient
Age group	0.817
Past Surgical History	0.52
Past history of Dentist visit	0.335
Primary Health Care Department	0.304
History of tattoo or cupping	0.235
Past history of jaundice	0.223
Gender	0.203
History of chronic illness	0.119
History of jaundice in the contacts	-0.06
History of blood transfusion	0.039

Note: * Coefficient: Pooled within-groups correlations between discriminating variables and standardized canonical discriminant functions

From the list of 10 predictors used in the discriminant model only age was enough to yield a statistically significant discriminant model. The model had a zero predictive accuracy for positive anti-HEV and 100% accuracy for negative group. The overall prediction accuracy of the model was 98.5%. Although, the overall performance of the model based on age alone was very good, it is almost useless in predicting positive HEV. This is an important disadvantage, since such a model is expected to screen for possible cases in the first place, Table 7.

**Table 7:** Forward selection method for Discriminant analysis model with selected independent variables and results of serum anti-HEV antibodies test as the dependent variables

Selected independent variables	Coefficients used in the discriminant equation
Age group (coded as 1 for <5 yrs, 2 for 5-14 yrs, 3 for 15-44 yrs and 4 for 45+ yrs)	1.293
(Constant)	-2.972

	**Discriminant score at group centroids**
Negative Anti-HEV antibodies	-0.008
Positive Anti-HEV antibodies	0.529
Critical value	0.2605

P (model)<0.001

Discriminant score (D) = -2.972 + [1.293 × Age group (coded as 1 for <5 yrs, 2 for 5-14 yrs, 3 for 15-44 yrs and 4 for 45+ yrs)]

**Table:** 

Accuracy of prediction for negative test results	100%
Accuracy of prediction for Positive test results	0%
Overall Accuracy of prediction	98.5%

When the value of age is entered in the equation the discriminant score (D) is calculated. If the resulting D score for a subject is higher than the critical value of 0.2605 then we predict a positive HEV case, while a D score lower than the critical value predicts a negative HEV status. The more extreme is the calculated value of D the higher our confidence in the prediction accuracy. Based on the present model, one can exclude the presence of HEV antibodies when age is < 15 years with high confidence, while an age 15+ will only increase the probability of having HEV with very low confidence, table 7.

As shown in Table 8, it is obvious that anti-HAV positive antibodies were more frequent in autumn season.

**Table 8:** The rate of anti-HAV and HEV antibodies positive tests among suspected cases by seasonal variation

	Total suspected cases	Anti-HAV antibodies		Anti-HEV antibodies	
Seasonal distribution	N	N	%	N	%
Summer	394	169	42.9	7	1.8
Winter	572	374	65.4	6	1
Spring	408	161	39.5	5	1.2
Autumn	301	148	49.2	2	0.7

## 4. Discussion

Viral hepatitis is one of the most important infectious diseases in the world. In Iraq, the epidemiology of viral hepatitis is different according to virus type. A study done in 2006 in Iraq (Ataallah M. Turky, 2011) showed that the prevalence of anti HAV IgG is 95% and of anti HEV IgG is 20%. Baghdad is the capital city of Iraq. It is divided by the Tigris River into north part (Alrussafa) and south part (Alkarkh). Each part composed of about 7-8 primary health care sectors which are in turn composed of primary health care centers. The total number of primary health care centers in Alkarkh is (98) and in Alrussafa (93) (Center section, primary health care office, Ministry of Health, Iraq, 2010). The present study has evaluated the acute stage antibody reaction to Hepatitis A & E measured by IgM titre.

The total number of the primary healthcare visitors in both sectors of Baghdad city during 1 year was (7576372) of them **2692 (35.5 per 100000)** were suspected cases of acute viral hepatitis. This figure is slightly higher than what was found by Hosker et al. (1988) in Newcastle. It was much higher than what was found in Egypt (Meky, 2006) and in residents of Rochester, Minnesota (Osmon, 1987). However, it was much lower than what was found in India (Singh, 1997) and in New Zealand (Lane, 1987). Of the suspected cases, only **1332 (49.5% of the suspected cases or 17.6 per 100000 of the consultations)** tested positive for one of the viral hepatitides. This was much lower than what was found in India (Singh, 1997). Although the results of blood testing for hepatitis A & E are less than expected but we think that the signs and symptoms are intermingled with signs and symptoms of other illnesses similar to viral hepatitis like flue, epigastric discomfort and other causes of acute hepatitis like CMV, Epstein bar viruses and others. Differences in probability of testing positive for one of the viral hepatitis markers among suspected (jaundice) cases in developed countries with high prevalence of infectious disease are attributed to differences in case definition of suspected cases in addition to differences in prevalence / incidence rates of viral hepatitis between countries.

In the present study, only 49.5% of the suspected cases had a positive viral marker. This figure is much lower than what is expected based on international experience described by Coppola et al. (1996) who assumed that 90% of the suspected cases will be caused by one of the viral hepatitides. Hepatitis A was supposed to form 40-60% which is nearly close to what was found in the current study. Hepatitis E was supposed to form around 5% (Coppola, 1996). This discrepancy might be attributed to wide range of inclusion criteria and improper application of case definition of suspected cases of viral hepatitis used in primary health care settings.

The positivity rate for Hepatitis A among suspected cases was 44.8%. This finding was slightly higher than what was reported in Brazil (Villar, 2002), in Japan (Yano, 2010) and southern Saudi Arabia (Ayoola, 2001). However, it was nearly close to what was found by another study in Saudi Arabia (SF., 1987) and in Portugal (Lecour, 1986). Hepatitis A is ubiquitous in nature and its mode of transmission is mostly by feco-oral route. Despite the higher number of the primary health care centers and jaundiced patients in Alrussafa side was higher than that of Alkarkh side, a patient from Alkarkh side has increased the risk of testing positive for Anti HAV by almost 1.5 compared to Alrussafa. The infrastructure and performance of municipal services and sanitation is different between the 2 sectors of Baghdad. Generally, the incidence of Hepatitis A is closely related to the socioeconomic conditions of sanitation and hygiene (Villar, 2002).

It has been found that the infection with acute hepatitis A and E varies with age as Hepatitis A was more common in less than 14 years with nearly 64% for each of those less than 14 years old. This predilection to pediatric age group is highly related to the endemicity of Hepatitis A which is in turn related to the hygienic conditions. In highly endemic areas with Hepatitis A, where there is low standard of hygiene, safe food and water, the infection usually occurs early in life causing long lasting immunity (Bell, 2005). These findings were similar to what is found by Sanaa et al. (2010) Bener et al. (2009). In addition, during war, there was huge destruction of the infrastructure including safe water, safe disposal of both sewage and garbage. Also, there was destruction of electricity which had made great possibility to get infection with hepatitis A and E all over the country. However, we don’t expect that hepatitis A occur in an epidemic form because is well known that hepatitis A is hyper prevalent in Iraq (95%), so the people at risk are less than 5% of the total population distributed all over the country but as far as hepatitis E is concerned, it might appear very clearly because the prevalence is 20% (Turky, 2011) and the people at risk are 80% of the population.

In this study, acute Hepatitis A was slightly higher in men compared with women. One possible explanation for that male preponderance is that males in the Iraqi society are usually more exposed to outdoor acquired infections and tolerate health risky behaviors more than women (Faleh, 2008). Similar findings were also mentioned in a surveillance report in United States (Department of Health, NYSDOH BCDC Surveillance Unit) and by Kamal et al. (2010) in Egypt in a survey of Acute hepatitis from 2001-2004. However, it has been reported that men and women are subject to the same frequency of infection, irrespective of age or race and seasonal or regional factors (Kuntz, 2008).

Presence of other factors (history of blood transfusion, history of chronic illness, history of surgery, history of tattooing & cupping, dentist visit, history of the same illness in the contacts, previous history of the illness) has not shown increase in the occurrence of Hepatitis A as they are mainly considered as possible risk factors for hepatitis B and C due to exposure to contaminated blood and body fluids in addition to invasive instruments without proper sterilization. In contrast, their absence may greatly support that the cause of jaundice is Hepatitis A and excludes hepatitis B or C. However, they have been looked for here as the survey included testing for hepatitis B and C as well in suspected acute viral hepatitis patients. This finding will lead the attention to be focused on the water and food supply sources as the main reason for the occurrence of both hepatitis A and Hepatitis E.

The rate for Hepatitis E was 1.6%. Being in Alrussafa side has increased the risk of testing positive for Anti HEV by almost 1.5 than being in Alkarkh. This finding was much lower than what was found in India (Lakshmi, 2011), Zimbabwe (Paolo, 1984) and Saudi Arabia (Ayoola, 2001). However, it was nearly similar to what was found in Egypt (Meky, 2006) and in Taiwan (Chu, 1999). Hepatitis E is mainly transmitted by cross contamination of drinking water with sewage. The outbreak of Hepatitis E that had happened in 2006 in Alsadr city which is located at Alrussafa side is a clear example as it had been found that it was caused by cross contamination of the water supply with sewage (Al-Nasrawi, 2010).

The HEV infection preferentially reaches teenagers and young adults (Pawlotsky, 2001). In this study, Hepatitis E was more common in young adults (15-44 years). This seems paradoxical for an enteral infection transmission, in which exposure is theoretically the same for all people subject to the terms of hygiene (Pawlotsky, 2001). It is possible that HEV infection is usually anicteric and goes unnoticed in children. These findings agree with the results found by Clark and Pelosi (2008), Kamal et al. (2010), Alavian (2007), and Ataallah et al. (2011).

The rate of occurrence of hepatitis E was not significantly higher in men than women. Although it was not significantly higher, it could be explained by a greater exposure of men in their professional and social activities (Pawlotsky, 2001). It has been found in other studies that it is higher in men than women (Kamal, 2010), (Pawlotsky, 2001) and (Said, 2009), while other studies have shown no clear gender predilection for the occurrence of hepatitis E (Shamsizadeh, 2009).

Similar to what has been found in Hepatitis A, presence of other factors (history of blood transfusion, history of chronic illness, history of surgery, history of tattooing &cupping, dentist visit, history of the same illness in the contacts, previous history of the illness) has not shown increase in the occurrence of Hepatitis E. In contrast, their absence may greatly support that the cause of jaundice is Hepatitis E and excludes hepatitis B or C due to same reasons mentioned for the hepatitis A.

Regarding seasonal variation of the occurrence of different types of hepatitis, it has been found that Hepatitis A was more common in winter (65.4%) and autumn (49.2%) which if linked with the most common age groups affected, it could have been due to the beginning of the studying semesters at the schools. This will lead to gathering of the children and as the virus is ubiquitous so this will increase the exposure to the virus. On the other hand, Hepatitis E was more common in summer (1.8%) which may be due to the increase exposure to the unsafe water supply.

## 5. Conclusions

1- This study has shown that the incidence of suspected acute viral hepatitis was (35.5 per 100000) of the total patients visiting primary health centers in Baghdad.
2- Testing suspected cases for viral hepatitis markers showed that (1206) 44.8% were due to Hepatitis A and (42) 1.6% were due to hepatitis E. This is considered low and might be attributed to wide inclusion criteria of suspected cases and the similarity of the constitutional symptoms with other diseases.
3- Factors that were able to predict positive Anti HAV IgM were (younger age group, negative history of cupping or tattooing, negative past history of jaundice). Male gender was the least important predictor of a positive case for anti HAV IgM. Prediction accuracy was 70.8%.
4- Of the factors studied, only age was able to predict a positive Anti HEV IgM in those more than 15 years with accuracy of 98%.

## Recommendations

1- The low rate of the confirmed cases of viral hepatitis among the suspected cases requires strict application of standard case definition, in addition to raising the awareness of health staff about the suspected acute cases of viral hepatitis in general.
2- Clinical diagnosis of acute viral hepatitis must be confirmed by serology to detect all the types of causative viruses (A, B, C and E), since presence of certain predictors cannot be considered as a rule for expecting the type of the virus. Therefore, sustainable availability of the diagnostic kits of all types should be maintained in health care centers.
3- As occurrence of Hepatitis A and hepatitis E is mainly linked to contaminated water and food supply, therefore, controlling them requires the collaborating sectors to work together to supply safe water and safe sewage disposal.
4- Health education is a cornerstone in prevention and control of the disease. Use of media and new communication channels help a lot in raising awareness towards the disease and the prevention methods.
